# Superhydrophilic Antifog Glass and Quartz Induced by Plasma Treatment in Air

**DOI:** 10.3390/nano15141058

**Published:** 2025-07-08

**Authors:** Huixing Zhang, Xiaolong Fang, Xiaowen Qi, Chaoran Sun, Zhenze Zhai, Longze Chen, He Wang, Qiufang Hu, Hongtao Cui, Meiyan Qiu

**Affiliations:** 1 School of Mechanical Engineering, Tianjin Sino-German University of Applied Sciences, Tianjin 300350, China; 830240@163.com (H.Z.); qiumeiyan666@126.com (M.Q.); 2 Department of Materials Science, School of Civil Engineering, Qingdao University of Technology, Qingdao 266520, China; fangxiaolong0929@163.com (X.F.); qixiaowen150@126.com (X.Q.); scr2393025128@163.com (C.S.); sw1607794385@163.com (Z.Z.); 19863731932@163.com (L.C.); 15948486077@163.com (H.W.); 15764280210@163.com (Q.H.)

**Keywords:** antifog, plasma treatment, surface structure, superhydrophilicity

## Abstract

Fogging on glass poses a severe challenge in daily life, potentially even becoming life-threatening during driving and surgery; therefore there is a need for antifog surface structures. Fabricating superhydrophilic surfaces has been one of the major solutions to the challenge. Conventional direct thermal annealing glass in a furnace at 900 K for 2 h led to superhydrophicity but failed to produce superhydrophilicity on quartz. Meanwhile, it degraded transmission and was low throughput. This study developed a programmed fast plasma treatment of planar soda-lime glass and quartz in air, applied for only a few seconds, that was able to fabricate superhydrophilic surfaces. The process led to a 0° contact angle without sacrificing transmission, a result unreported before. The plasma treatment covered a whole 30 × 30 cm^2^ substrate in only approximately 5 s, resulting in superhydrophilicity, which has rarely been reported before. This simple yet controllable process has great potential for further scale-up and practical applications.

## 1. Introduction

Glass has been extensively utilized in the automotive, photovoltaic, and medical industries. However, fogging on glass surfaces commonly occurs when exposed to sudden changes in temperature and humidity, posing a significant challenge. During fogging, condensed water droplets scatter light, impairing visibility and hindering proper function—potentially endangering lives in critical scenarios such as driving and surgical operations [1,2,3,4]. Quartz, which is widely employed in high-end applications such as electronics and optics, also needs high levels of optical clarity [5]. Therefore, antifogging performance is highly desirable in these materials across these fields. The two primary strategies for achieving antifogging properties are surface chemical modification and the enhancement of surface roughness, which promote either superhydrophilicity or superhydrophobicity [6,7,8,9]. Superhydrophobic surfaces have received significant attention for a wide range of applications, including corrosion resistance, fouling prevention, self-cleaning, oil–water separation, viscous drag reduction, and anti-icing [10]. In contrast, practical applications have demonstrated that superhydrophilic surfaces often outperform their superhydrophobic counterparts in terms of self-cleaning efficiency and reducing dust cementation [11,12,13]. Besides these factors, superhydrophilic methods require substantially less water, as a thin water film is sufficient to remove surface contaminants. In comparison, superhydrophobic surfaces typically rely on a much larger volume of water to encapsulate and carry away debris [14]. Upon condensing on a super hydrophilic surface, water droplets spread out instantly to form a water film, preventing light scattering from the droplets. Achieving superhydrophobicity typically requires complex fabrication techniques that are often constrained to laboratory-scale production [10,15,16]. Consequently, the development of superhydrophilic surfaces has become a key approach for practical antifogging applications [6,17].

Simple and direct methods for fabricating superhydrophilic surfaces are gaining increasing research attention [18,19]. Thermal processing protocols (900 K for 2 h in a furnace) have been shown to convert hydrophilic soda-lime glass (contact angle ~54°) into a superhydrophilic state (contact angle ~1°). However, this approach is energy-intensive and characterized by low productivity [18]. The observed enhancement in wettability arises from the synergistic effects of surface chemical redistribution—namely, increased concentrations of Na^+^ and Ca^2+^ due to oxide segregation and surface migration—and the associated evolution of nano-roughness, which increased from 0.74 nm in the untreated sample to 1.35 nm after annealing. Na_2_O and CaO are known to adsorb water, thereby enhancing surface hydrophilicity [18]. Crucially, identical thermal treatment did not produce similar wettability changes on fused quartz or crystalline silica [18], likely due to their distinct network structures and cation mobility limitations. Achieving superhydrophilicity on quartz thus remains a challenge. Plasma treatment, which generates a high density of energetic and reactive radicals, has been widely employed for surface modification, cleaning, and wettability alteration [19]. These highly reactive species may interact with the quartz surface to modify its wettability.

To enhance the bonding between glass and other materials, low-temperature plasma treatment has been developed as an effective approach to improve the wettability of glass surfaces [19]. This treatment increases the surface oxygen content while reducing the carbon content. Oxygen tends to form polar functional groups (e.g., –OH), whereas carbon is more likely to form nonpolar bonds (e.g., C–C and C–H). Yang et al. demonstrated that the enhanced hydrophilicity of the glass surface originates from the predominance of polar functional groups over nonpolar moieties, with wettability highly correlated with polar group concentration [20]. In their study, plasma treatment was conducted under a constant oxygen flow of 40 sccm, and a treatment duration of one minute yielded the lowest water contact angle (WCA) of 2.6°. However, extended treatment times led to an increase in WCA, which was accounted for by the over etching of polar groups and exposure of nonpolar groups.

Plasma technology, recognized as an efficient and environmentally conscious surface treatment method, is capable of significantly altering the properties of material surfaces [21]. In comparison to conventional wet chemical or mechanical treatments, plasma processing offers advantages such as being a dry, rapid, and controllable technique [22]. Notably, air plasma generates a diverse array of high-energy reactive species, including reactive oxygen species (ROS) such as atomic oxygen (O), ozone (O_3_), and hydroxyl radicals (·OH), as well as reactive nitrogen species (RNS) like atomic nitrogen (N) and nitrogen oxides (NOx), alongside charged particles and ultraviolet (UV) radiation [23]. These highly reactive entities engage in complex physicochemical reactions with material surfaces, facilitating both surface cleaning and activation.

Initially, the bombardment of high-energy electrons within air plasma leads to the scission of chemical bonds in organic contaminant molecules adsorbed on glass and quartz surfaces, causing their fragmentation into smaller, volatile species and consequently achieving surface cleaning [24]. Subsequently, reactive oxygen species, acting as potent oxidizing agents, can oxidize residual organic contaminants on surfaces, transforming them into readily removable small molecules such as carbon dioxide and water [25]. For instance, hydroxyl radicals (·OH), characterized by their exceptionally high oxidation potential, effectively eliminate a wide range of organic pollutants [26].

Furthermore, the reactive species present in air plasma can interact with Si–O bonds on the surfaces of glass and quartz, leading to the incorporation of hydrophilic oxygen-containing functional groups, such as silanol groups (Si–OH) and oxygen vacancies [27]. The increased presence of these polar groups significantly enhances surface hydrophilicity, resulting in a reduction of the water contact angle and establishing a chemical basis for achieving superhydrophilicity [28]. Consequently, the application of air plasma to treat glass and quartz leverages the synergistic effects of surface cleaning and activation mechanisms to effectively improve their wetting properties.

Plasma treatment, owing to its efficiency, environmental friendliness, and ability to modify surfaces at low temperatures, has become a crucial method for enhancing the surface properties of materials such as glass and quartz. The characteristics of the plasma source are paramount to its treatment efficacy and are determined by factors including its discharge type and excitation method [29]. In recent decades, atmospheric pressure plasma jets (APPJs) have garnered significant attention as a promising plasma source [30]. APPJs offer the distinct advantage of providing spatially unbound and electrode-unconfined plasma plumes, enabling the treatment of complex geometries or precise localized areas far from the electrodes. The superiority of APPJs lies in their ability to achieve uniform discharge characteristics akin to low-pressure glow discharges under specific operating conditions. Furthermore, APPJs boast additional benefits such as a low gas temperature (suitable for heat-sensitive materials), high concentrations of reactive species, no vacuum requirement, and scalability for larger-area processing, offering unique advantages over traditional plasma sources for material surface modification.

For the surface modification of glass and quartz, the precise control of APPJ treatment conditions—including power, scanning speed, the distance between the plasma nozzle and the substrate, and gas composition—is critical for determining the final surface properties, such as hydrophilicity and antifog performance. These parameters collectively influence the material’s surface chemical composition and surface morphology.

Specifically, power directly affects the generation efficiency and concentration of active species, ion bombardment intensity, and plasma temperature. Excessively high power may lead to the over-etching of the surface polar groups and exposure of nonpolar groups, potentially compromising hydrophilicity and long-term durability [19].

Scanning speed (or treatment time) dictates the total energy and active species flux to which the material surface is exposed. An excessively slow scanning speed is equivalent to excessively high power. To achieve optimal hydrophilicity, the treatment speed must be optimized to balance the adequate introduction of hydrophilic groups with the formation of an appropriate surface bond and structure [31].

In this study, APPJs were employed to treat soda-lime glass and quartz substrates, aiming to fabricate super-hydrophilic antifog surfaces. The processing speed was optimized for this research, with a setting power of 1250 W and a constant plasma nozzle and substrate distance at 40 mm. This paper elaborates on how these treatment parameters influence the microstructure and chemical composition of glass/quartz surfaces, subsequently elucidating their mechanisms for modulating hydrophilicity and antifog performance. Air instead of O_2_ was adopted for the plasma treatment in this study, resulting in a WCA of 0°, a result not reported before. Meanwhile, treatment of a large 900 cm^2^ sample in approximately 5 s led to WCA of 3.6°. Such rapid processing of large sample for superhydrophilicity has rarely been reported. The regeneration of superhydrophilicity by plasma re-treatment was demonstrated here for the first time. Compared to traditional methods, particularly superhydrophobic treatments, the plasma method is more simple, environmentally friendly, scalable, programmable, and controllable, making it a promising technique for practical and large-scale applications.

## 2. Experiments

### 2.1. Materials and Methods

**Figure 1:**
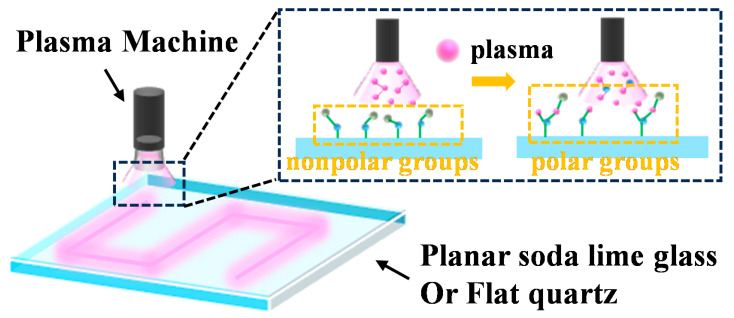
The schematic of the plasma treatment process.

Soda-lime glass and quartz substrates were cleaned sequentially by sonication in acetone, isopropanol, and deionized water for 3 min each. The cleaned substrates were blow-dried under compressed air and then transferred to a platform ready for plasma treatment. A computer-controlled plasma cleaner (E520X, Shenzhen Good Art Automation Technology Ltd., Co., Shenzhen, China) was employed to process the substrates directly in ambient air, following a programmed scanning path. The treatment was conducted with a power output of ~1250 W (230 V, 9 A), a focal spot size of 15 mm, a distance between the plasma nozzle and the substrate of 40 mm, and varying the scanning speed from 0.05 to 1 m/s. Complete coverage of a 30 × 30 cm^2^ substrate could be achieved within 5 s. Most of the substrates used had sizes of 5 × 5 cm^2^; the large substrate is for a demonstration of scalability. A schematic illustration of the process is shown in Figure 1.

### 2.2. Characterization

The surface chemical composition of the samples was analyzed using X-ray photoelectron spectroscopy (XPS, Thermo Scientific K-Alpha, Shanghai, China). Surface wettability was evaluated by measuring the WCA using a goniometer (DALUE Standard-2C, Zhuhai, China); at least three measurements were taken on each sample to obtain an average. A micro-pipette was used to dispense 1 µL water droplets onto the sample surface, and images were captured using a CCD camera. Surface morphology was characterized using atomic force microscopy (AFM, Bruker Dimension Icon, Berlin, Germany). Optical transmittance in the wavelength range of 400–1000 nm was measured using a UV-Vis-NIR spectrophotometer (Lambda 1050, PerkinElmer, Shelton, CT, USA).

### 2.3. Antifogging Test

A hot water kettle was used to generate steam, and each sample was individually exposed to the steam at a distance of 40 mm above the outlet for 10 s. The antifogging performance of the samples was evaluated by photographing them against a consistent background displaying Beijing time according to the official website of the Greenwich Observatory.

## 3. Results and Discussion

**Figure 2:**
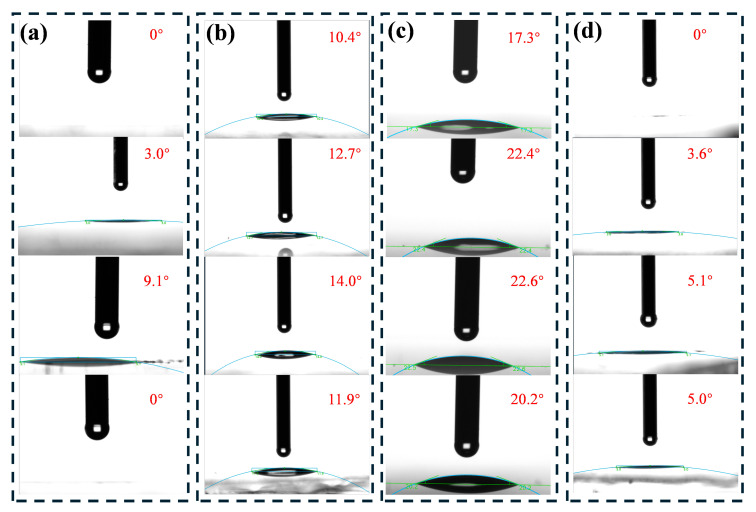
Contact angles of samples treated at different processing speeds and their evolution during storage in the laboratory and after a second plasma treatment using identical parameters as the initial treatment. Processing speeds, from top to bottom, are 30, 50, 80, and 100 mm/s. (**a**) Immediately after the first plasma treatment; (**b**) after one day of storage indoors; (**c**) after three days of storage indoors; and (**d**) immediately following the 2nd plasma treatment.

The WCA of the sample was observed to increase with increasing plasma treatment speed, as shown in Figure 2. The maximum WCA was 9.1°, which was achieved on the sample surface at a velocity of 0.08 m/s. However, when the velocity was further increased to 0.1 m/s, the WCA on the sample surface decreased to 0°. However, the WCA increased again while following a decreasing trend for treatment speeds above 0.1 m/s, with values between 3 and 5°, as shown in Figure S1. A treatment speed of 1 m/s covered an entire 900 cm^2^ substrate in approximately 5 s and achieved a WCA of 3.6° (the plasma treatment process is demonstrated in Videos S1 and S2). This is the first report of such a large sample being processed for superhydrophilicity in such a short time. Due to airborne hydrocarbon deposition and accumulation on the sample surface [32], the WCA gradually increased, reaching 10.4–14.0° after one day storage in the laboratory and further rising to 17.3–22.6° after three days of indoor storage. Following a second plasma treatment, the glass surface effectively reverted to its superhydrophilic state, which was fairly efficient and first reported. It is important to note that fluorosilane was used in the laboratory [33], meaning the laboratory is a difficult environment in which to maintain superhydrophilicity.

Oxygen plasma treatment was previously used to improve surface wettability, achieving a WCA of 2.6° with one minute of treatment [19]. In this study, air instead of oxygen was employed and a lower WCA of 0° was achieved. Meanwhile, extended treatment times led to an increased WCA for oxygen plasma treatment, which in our case enhanced superhydrophilicity. This contrast demonstrates that air plasma not only outperforms oxygen plasma in wettability enhancement but also offers an original and effective alternative. The process was implemented using a commercial plasma system, with the surface modification method developed in-house. This approach is readily scalable to larger glass substrates, as demonstrated by the contact angle measurement shown in Figure S2. Also, the treated glass has a uniform surface finish and excellent antifogging properties, as evidenced by Videos S1–S3. With appropriately scaled equipment, the method can be extended to even larger substrates. This work serves as a proof-of-concept study, highlighting the feasibility of practical and scalable application of the technique.

**Figure 3:**
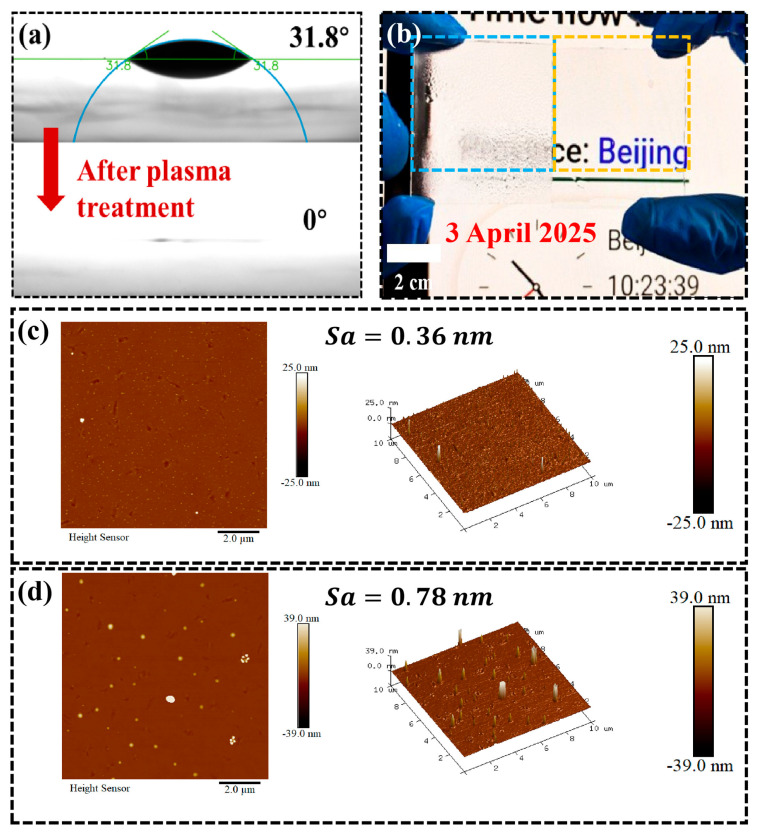
(**a**) WCA of planar soda-lime glass before and after plasma treatment. Antifog performance of planar soda-lime glass before and after plasma treatment (**b**). The plasma treated sample was yellow framed, the reference blue framed. AFM images of planar soda-lime glass before (**c**) and after (**d**) plasma treatment.

To validate the results and investigate the underlying mechanism, a control experiment consisting of a plasma-treated sample and reference glass was conducted. Figure 3a shows a WCA of 31.8° for the reference glass, which decreased to 0° within 0.4 s after plasma treatment. This is the first time this has been reported. Figure 3b presents the antifog performance of planar soda-lime glass before and after plasma treatment. The plasma-treated sample exhibited excellent antifog properties. The sharp reduction in the WCA and enhanced antifog performance was accounted for by changes in the surface roughness and chemical composition. Figure 3c,d show AFM images of soda-lime glass before and after plasma treatment. The original reference had a root mean square (RMS) roughness of 0.37 nm, with a height range of −25 to 25 nm. In comparison, the plasma-treated sample showed an increased roughness of 0.79 nm and a broader height range of −39 to 39 nm, characterized by distributed nanoscale protrusions. The protrusions may be caused by phase separation and the overflow of some oxide to the surface, as previously reported [18]. This accounted for the increased roughness of the treated sample, while there was no clear feature structure on the smooth reference glass.

**Figure 4:**
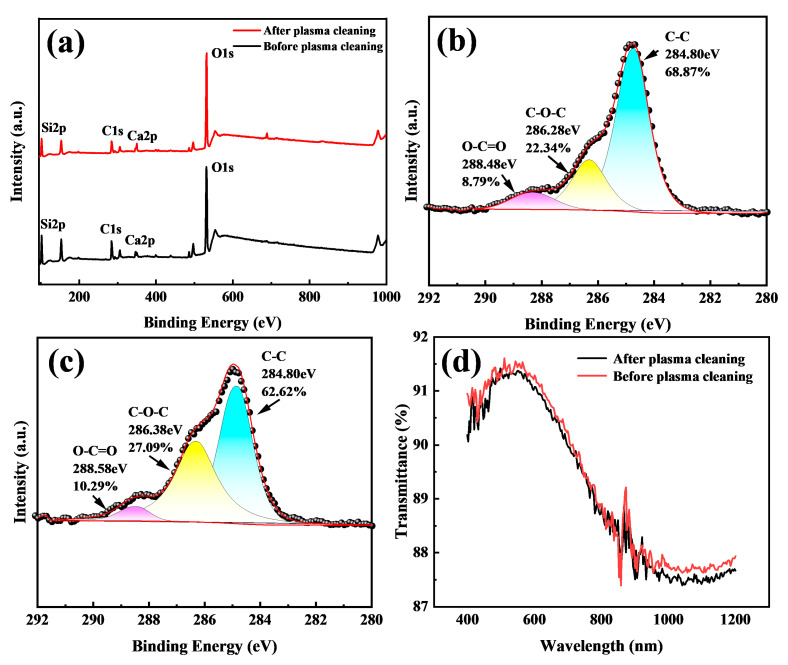
(**a**) XPS spectra of planar soda-lime glass before and after plasma treatment. (**b**) C1s region before plasma treatment. (**c**) C1s region after plasma treatment. (**d**) Transmission spectra of planar soda-lime glass before and after plasma treatment.

Additional to the surface roughness enhancement, surface chemical changes may play a significant role in superhydrophilicity [18]. Figure 4a shows the full XPS spectra of soda-lime glass before and after plasma treatment. The full spectra shows that the primary surface elements are O, Si, C, and Ca. Si and Ca were components of glass and from the substrate, while C might originate from the deposition organic contaminates from the environment [34,35,36]. Notably, plasma treatment significantly increased the intensities of the Ca2p and O1s peaks, with the calcium content rising from 1.1% to 1.5% and the oxygen content increasing from 42.22% to 54.12%. This increase may be attributed to phase separation and oxide migration from the bulk glass to the surface. Given that CaO is known to adsorb water molecules [18], this contributes to enhanced hydrophilicity and a reduced WCA. Conversely, the carbon content decreased following plasma treatment, further enhancing surface hydrophilicity.

The C1s peak at 284.8 eV was used as a reference for calibration. Figure 4b,c show the high-resolution C1s spectra, which consist of three primary components at 284.8 eV (C–C), 286.0 eV (C–O–C), and 288.5 eV (O–C=O). Only the C–C bond is nonpolar. The improved hydrophilicity can be attributed to the increased proportion of polar functional groups relative to nonpolar ones [20]. Specifically, the total carbon content decreased from 30.28% to 20.13% after plasma treatment. Meanwhile, the relative concentrations of the polar groups C–O–C and O–C=O increased from 22.34% and 8.79% to 27.09% and 10.29%, respectively, while the nonpolar C–C component decreased from 68.87% to 62.62%. These results suggest that the plasma treatment preferentially removed nonpolar organic species.

According to the literature, C1s signals in XPS spectra typically originate from the adsorption of organic contaminants from the environment during storage periods and transport prior to measurement [34,37]. Plasma treatment is known to effectively remove these surface-bound organics, resulting in a superhydrophilic surface. However, re-adsorption of airborne organic species may occur before the XPS measurement is conducted. Due to this unavoidable ambient exposure, all measured surfaces tend to exhibit a detectable C1s signal. In some cases, especially when there is a long delay between treatment and measurement, the reduction in C1s signal intensity remains limited—often within 10%—even for high-performing samples [38]. By comparison, the plasma treatment employed in this study achieved a more pronounced reduction in surface carbon content, indicating improved effectiveness in mitigating surface contamination.

Both the increased surface roughness and enhanced polarity of the surface explain the wettability change and the antifog performance of the plasma-treated glass in comparison with the reference glass. According to the Wenzel model [39], increasing the roughness of an intrinsically hydrophilic surface enhances its hydrophilicity. However, when the roughness is exceedingly low, as in the present case, variations in roughness exert minimal influence on the overall wetting behavior. Therefore, the observed improvement in hydrophilicity can be primarily attributed to the chemical modifications induced by plasma treatment. As reported in the literature, an increase in surface polar functional groups and a reduction in nonpolar groups generally leads to enhanced hydrophilicity [19]. Our results show that plasma treatment increased polar contents of Ca and O species and reduced the nonpolar contents, such as C–C(H), substantially. These chemical alterations play a dominant role in improving the surface wettability.

As shown in Figure 4d, the optical transmission spectra of the treated and reference samples reveal negligible differences, with variations within only 0.1%, indicating that the plasma treatment does not compromise optical transparency.

**Figure 5:**
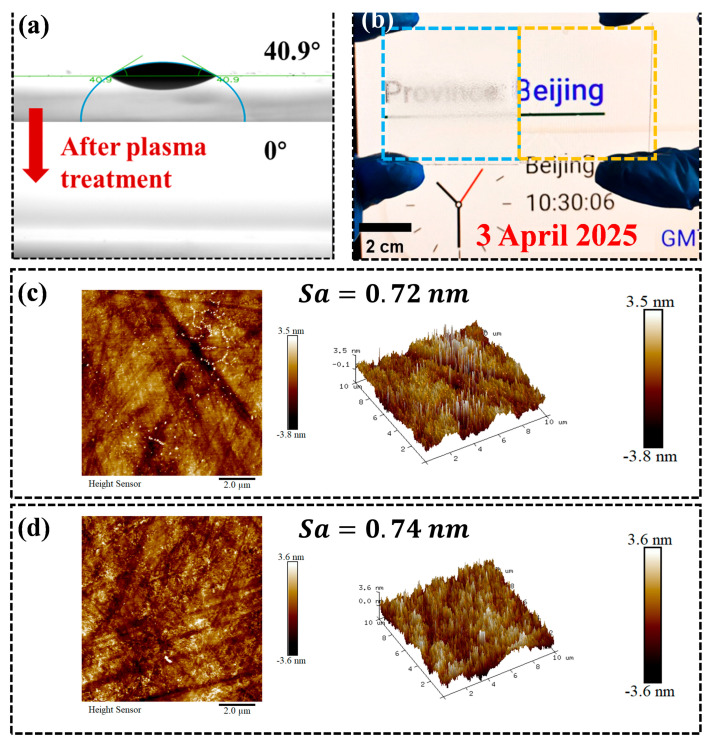
(**a**) The WCA of planar fuse quartz before and after plasma treatment. Antifog performance of planar quartz before and after flame treatment (**b**). The plasma-treated sample is yellow framed; the reference is blue framed. AFM images of planar quartz before (**c**) and after (**d**) plasma treatment.

**Figure 6:**
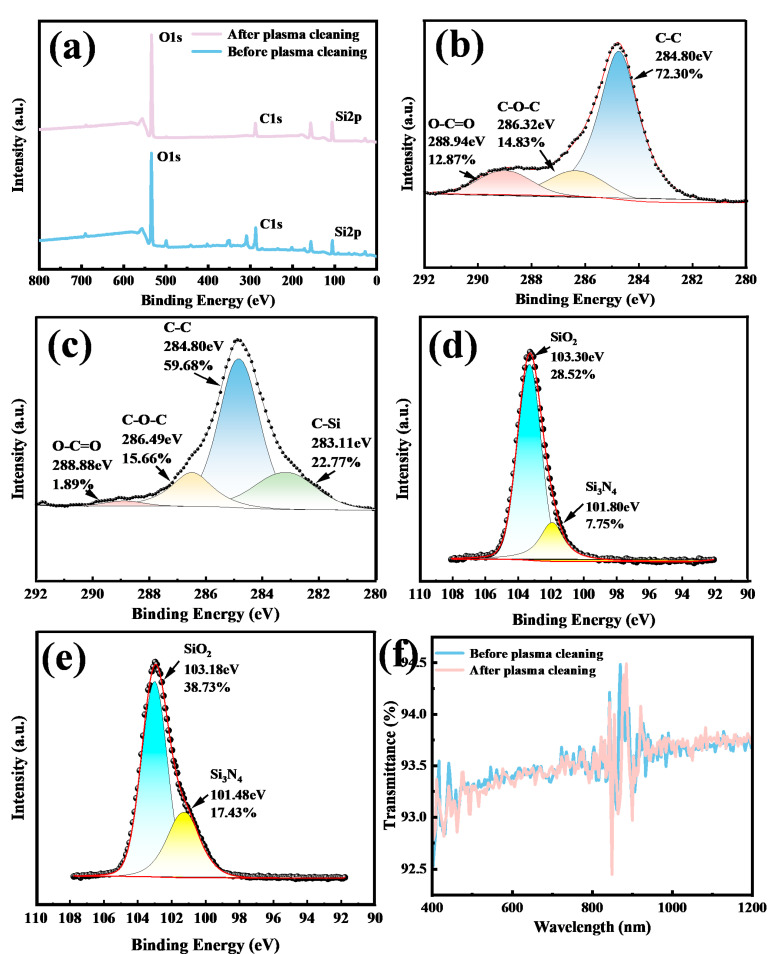
XPS spectra of planar quartz before and after plasma treatment (**a**). (**b**) C1s region before plasma treatment. (**c**) C1s region after plasma treatment. (**d**) Si2p region before plasma treatment. (**e**) Si2p region after plasma treatment. (**f**) Transmission spectra of flat quartz before and after plasma treatment.

Direct thermal annealing of quartz at 900 K for 2 h failed to produce a superhydrophilic surface [18]. In this study, plasma treatment was also applied to flat quartz substrates. The treatment reduced the WCA to 0°, with a spreading time of 0.61 s, which, to the best of our knowledge, has not been previously reported (Figure 5a). Furthermore, compared to the untreated reference, the plasma-treated sample demonstrated excellent antifogging performance, as shown in Figure 5b. Even if the roughness of the samples before and after the irradiation was almost the same, the treated sample exhibited a more wrinkled surface morphology in comparison with the relatively smooth reference. The chemical composition changes on surface played a critical role in altering the sample’s wettability. Figure 6a shows the full XPS spectra of quartz glass before and after plasma treatment. The spectra indicate that the primary elements present on the surface are C, O, and Si. A significant increase was observed in the oxygen content, from 46.89% to 51.79%, and in the silicon content, from 15.55% to 26.14%, indicating an enhanced presence of polar Si and O bonds. The increase in these polar components is associated with improved surface hydrophilicity [38].

Figure 6b,c display the high-resolution C1s spectra. Plasma treatment led to a reduction in the total surface carbon content from 31.91% to 21.39%. Correspondingly, the relative concentration of the polar O–C–O bonds increased from 14.83% to 15.66%, while that of O–C=O decreased from 12.87% to 1.89%. Meanwhile, the concentration of nonpolar C–C bonds dropped from 72.30% to 59.68%. Notably, an additional C–Si peak suggests that the plasma-activated surface became highly reactive and reacted with surface organics, forming stable polar bonds. Figure 6d,e show the high-resolution Si2p spectra. The results show that after plasma treatment, the content of polar bonds such as SiO_2_ and Si_3_N_4_ increased from 28.52% and 7.75% to 38.73% and 17.43%, respectively. These bonds were products of a reaction between plasma-generated highly reactive species (ROS, RNS) and the surface components. These compositional changes contributed to the reduced WCA and enhanced the wettability of the quartz surface. The compositional change was attributed to a plasma-induced surface reaction between the highly active plasma radicals, the surface components, and the deposited organics.

Figure 6f presents the transmission spectra of the samples before and after plasma treatment, revealing virtually no loss in optical transmittance.

## 4. Conclusions

We employed ultra-fast plasma treatment in air to produce superhydrophilic surfaces (WCA = 0°) on both flat glass and quartz. To our knowledge, the treatment of quartz in this way has not been reported previously. The plasma treatment significantly altered the surface morphology and composition of both materials. For soda-lime glass, the treatment induced phase separation and oxide overflow to the surface, resulting in increases in surface roughness and calcium concentration. In contrast, on quartz, the plasma treatment predominantly reacted with nonpolar species, removing more of the nonpolar content than the polar components. It also increased the polar contents of Si and O due to the reaction between highly active plasma species, surface components, and the deposited organics. These changes in surface structure and composition were key to achieving superhydrophilicity. The plasma-treated surfaces exhibited a contact angle of 0°, with negligible loss in transmission, a result that has not been previously reported.

Although the WCA increased to ~20° after three days of storage in the lab due to the adsorption of fluorosilane and other organic contaminants, superhydrophilicity was fully restored by reapplying the ultrafast plasma treatment, which covered a 900 cm^2^ sample in only ~5 s. This simple, rapid, and programmable process is highly scalable, making it suitable for large-area applications such as antifogging automotive windshields. Given that a full windshield (~2 m^2^) could be treated within 2 min, this technology presents a practical solution for enhancing driving safety by maintaining clear visibility under humid or foggy conditions. Moreover, the air-based plasma process is environmentally benign compared to conventional wet chemical methods and preserves optical transparency—an essential requirement for automotive and optical applications.

## Data Availability

All data are included in the paper.

## Supplementary Materials

The following supporting information can be downloaded at: https://www.mdpi.com/article/10.3390/nano15141058/s1, Figure S1: Contact angles of samples treated at different processing speeds and their evolution during storage at the laboratory and after a second plasma treatment using identical parameters as the initial treatment. Processing speeds from top to bottom are 150, 200, 500, and 1000 mm/s. (a) Immediately after the first plasma treatment; (b) after one day storage indoors (c) after three days storage indoors (d) immediately following the 2nd plasma treatment; Figure S2: Contact angles at different positions after plasma treatment of 10 cm × 10 cm glass; Video S1: Plasma treatment process covering 5 cm × 5 cm; Video S2: Plasma treatment process covering 30 cm × 30 cm; Video S3: Anti-fog effect of plasma treated glass.
