# Supplementary material for: Superhydrophilic Antifog Glass and Quartz Induced by Plasma Treatment in Air
Source: Nanomaterials (Basel). 2025 Jul 8;15(14):1058. doi: 10.3390/nano15141058 (PMC12298405; doi:10.3390/nano15141058)
Supplement: Supplementary file 1 [file nanomaterials-15-01058-s001.zip › nanomaterials-3740215-supplementary.pdf]

Superhydrophilic Antifog Glass and Quartz induced by Plasma Treatment in Air

Huixing Zhang [1], Xiaolong Fang [2], Xiaowen Qi [2], Chaoran Sun [2], Zhenze Zhai [2], Longze Chen [2], He Wang [2], Qiufang Hu [2], Hongtao Cui [2,*] and Meiyan Qiu [1]

[1] School of Mechanical Engineering, Tianjin Sino-German University of Applied Sciences, Tianjin 300350, China; 830240@163.com (H.Z.); qiumeiyan666@126.com (M.Q.)

[2] Department of Materials Science, School of Civil Engineering, Qingdao University of Technology, Qingdao 266520, China; fangxiaolong0929@163.com (X.F.); qixiaowen150@126.com (X.Q.); scr2393025128@163.com (C.S.); sw1607794385@163.com (Z.Z.); 19863731932@163.com (L.C.); 15948486077@163.com (H.W.); 15764280210@163.com (Q.H.)

* Correspondence: cuihongtao@qut.edu.cn

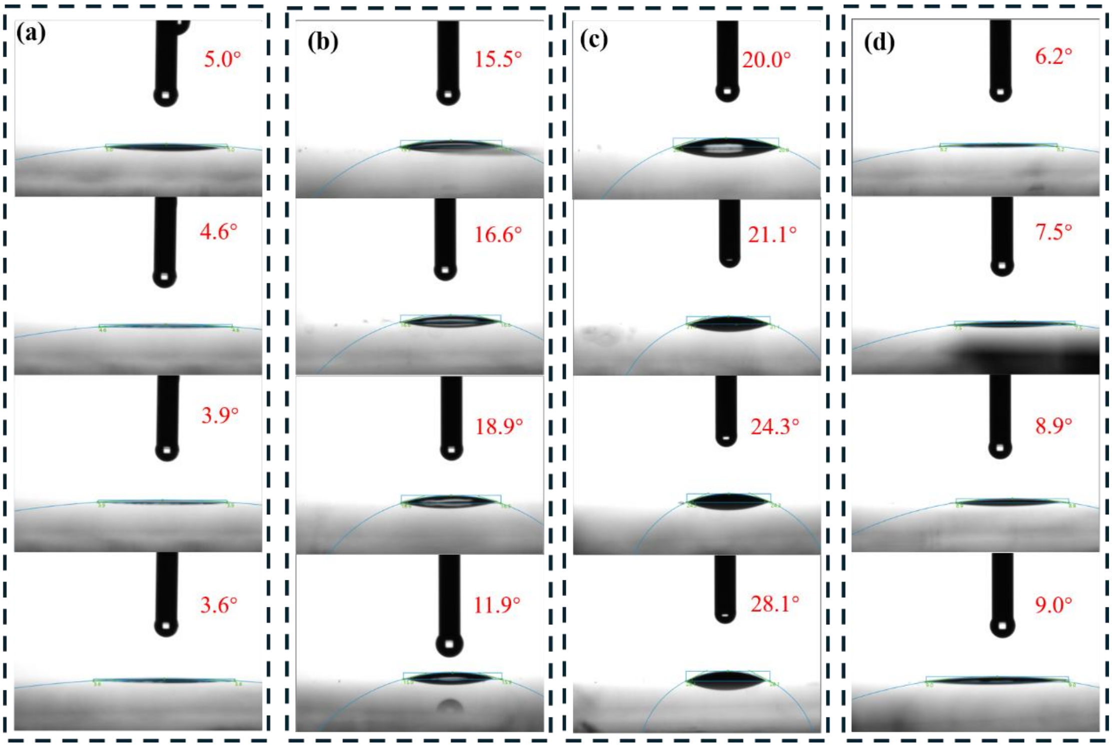

Figure. S1. Contact angles of samples treated at different processing speeds and their evolution during storage at the laboratory and after a second plasma treatment using identical parameters as the initial treatment. Processing speeds from top to bottom are 150, 200, 500, and 1000 mm/s. （a）Immediately after the first plasma treatment; (b) after one day storage indoors (c) after three days storage indoors (d) immediately following the 2nd plasma treatment.

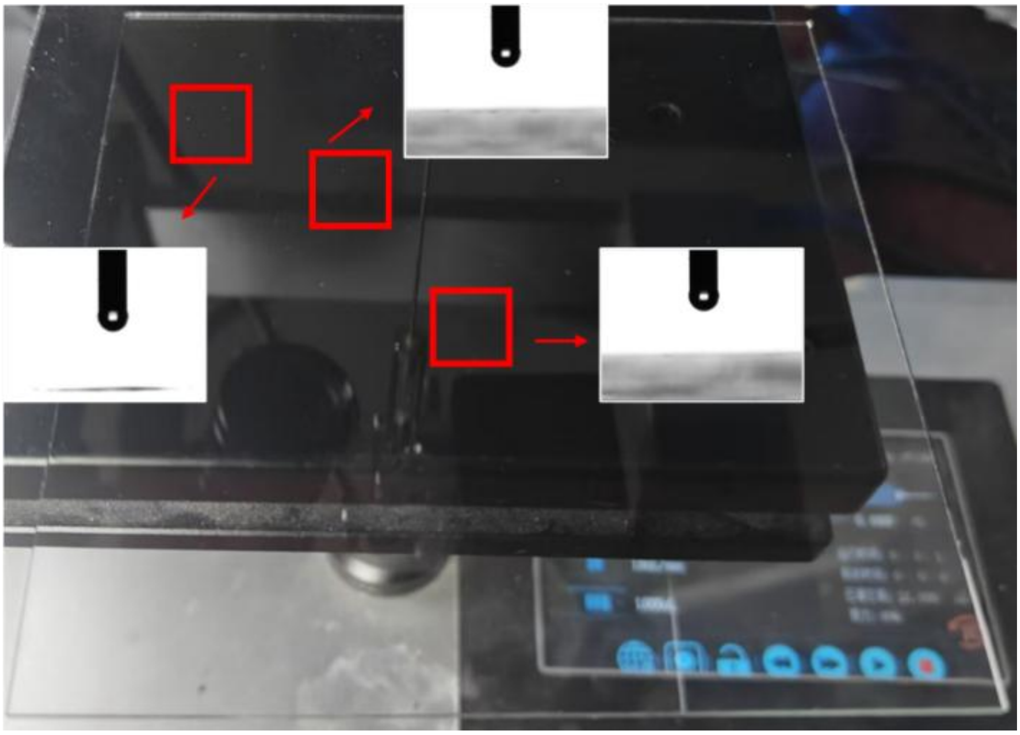

Figure. S2. Contact angles at different positions after plasma treatment of 10 cm×10 cm glass.
